# Ethylene and the regulation of plant development

**DOI:** 10.1186/1741-7007-10-9

**Published:** 2012-02-20

**Authors:** G Eric Schaller

**Affiliations:** 1 Department of Biological Sciences, Dartmouth College, Hanover, NH 03755, USA

## Abstract

Often considered an 'aging' hormone due to its role in accelerating such developmental processes as ripening, senescence, and abscission, the plant hormone ethylene also regulates many aspects of growth and development throughout the life cycle of the plant. Multiple mechanisms have been identified by which transcriptional output from the ethylene signaling pathway can be tailored to meet the needs of particular developmental pathways. Of special interest is the report by Lumba *et al*. in *BMC Biology *on how vegetative transitions are regulated through the effect of the transcription factor FUSCA3 on ethylene-controlled gene expression, providing an elegant example of how hormonal control can be integrated into a developmental pathway.

See research article http://www.biomedcentral.com/1741-7007/10/8

**Commentary:**

One of the amazing qualities of plants is their phenotypic plasticity. Consider, for example, how a pine tree will grow to a towering hundreds of feet in height in Yosemite Valley, but to only a gnarled few feet in height up near the timberline. This diversity of form, though originating from the same genotype, points to the degree to which plant growth and development can be modulated. Much of this control is mediated by a small group of plant hormones that include auxin, cytokinin, gibberellin, abscisic acid, brassinosteroid, jasmonic acid, and ethylene [1]. These are often considered 'classical' plant hormones because they were discovered decades ago; indeed, the presence of some was inferred over a century ago. Their early discovery is no doubt due in part to their general function throughout the life cycle of the plant. More recently, and in the remarkably short period of time since the advent of *Arabidopsis *as a genetic model, key elements in the primary signaling pathways of these plant hormones have been uncovered. The important question is no longer simply how are these hormones perceived, but how are the hormonal signals integrated into the control of particular developmental pathways? In pursuing such a question, Lumba *et al*. [2] have now uncovered a role for the plant hormone ethylene in regulating the conversion of juvenile to adult leaves. These new data, in combination with prior research implicating the plant hormones abscisic acid and gibberellin in this transition [3], form an important step in defining how a hormonal network regulates a key developmental process.

## Ethylene and plant development

Ethylene, for all the simplicity of its structure (C_2_H_4_), regulates many aspects of plant growth and development [4]. The phrase 'growth and development' may be one of the most commonly used scientific phrases (a Google search turns up over 17 million hits), but for our purposes it is worthwhile to disengage the terms 'growth' and 'development' from each other. Growth is quantitative and originates from an increase in the size and/or number of cells. Development is qualitative, and indicates that the cells have differentiated in some manner, whether at the subcellular, cellular, and/or tissue level. Changes in growth and development are often concurrent and both are under hormonal regulation.

Ethylene affects both the growth and development of plants [4]. In terms of growth, ethylene is most commonly associated with the regulation of cell size, often restricting cell elongation, but it can also regulate cell division. In terms of development, ethylene is most commonly considered an 'aging' hormone, as it accelerates and is sometimes required for such processes as ripening, senescence, and abscission. In this respect, mutations that affect ethylene production or perception can be considered heterchronic mutations as they alter the timing of a developmental process. However, ethylene not only affects the final stages of plant development, but also has regulatory roles on development throughout the life cycle of the plant. Ethylene stimulates root initiation in many plant species, controls the formation of root nodules in legumes, inhibits the formation of such storage organs as tubers and bulbs, promotes flowering in some species (but inhibits it in others), and induces the production of female rather than male flowers in cucurbits.

**Figure 1:**
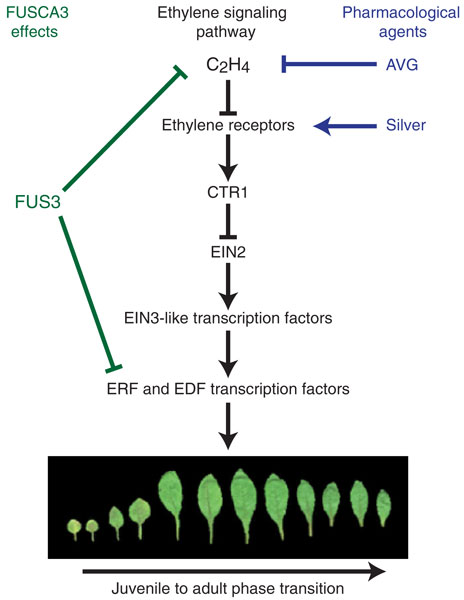
**Ethylene signal transduction and the control of juvenile to adult phase transitions in the leaf**. The pathway for ethylene signal transduction involves positive and negative regulators that culminate in transcriptional regulation by the EIN3-like family of transcription factors. Among the ethylene-responsive genes are some that encode additional transcription factors such as those of the ethylene response factor (ERF) and ethylene response DNA-binding factor (EDF) families. The pharmacological agents aminoethoxyvinylglycine (AVG) and silver can be used to inhibit ethylene responses through their ability to target ethylene biosynthesis or the receptors, respectively. One effect of ethylene is to stimulate the juvenile to adult phase transition of leaves. The transcription factor FUS3 negatively regulates the effects of ethylene on this developmental process. The juvenile to adult leaf morphology series shown is from Figure 3A in Lumba *et al*. [2].

Remarkably, this wealth of developmental effects is all mediated through the same primary signaling pathway [5]. The key elements in this signaling pathway (Figure 1) include the ethylene receptors, the Raf-like kinase CTR1, the transmembrane protein EIN2, and the EIN3-like family of transcription factors. This pathway involves both positive and negative regulators, such that ethylene response is actively repressed in the air and de-repressed in the presence of ethylene. Ethylene binding to its receptors serves to relieve the repression so that the EIN3-like transcription factors are activated to initiate the transcriptional response to ethylene. Significantly, among the genes induced by ethylene are several additional families of transcription factors - the ethylene response factor (ERF) and ethylene response DNA-binding factor (EDF) families - indicating that a transcriptional cascade acts downstream of the EIN3-like proteins.

As our understanding of the players involved in ethylene biosynthesis and signal transduction has increased, so has our ability to manipulate these to assess experimentally the role(s) that ethylene plays in plant development. As Archimedes once boasted: ΠΑ ΒΩ ΚΑΙ ΧΑΡΙΣΤΙΩΝΙ ΤΑΝ ΓΑΝ ΚΙΝΗΣΩ ΠΑΣΑΝ ("Give me a place to stand and with a lever I will move the whole world."). The pharmacological and genetic tools by which ethylene signaling can be modulated provide many such levers operating at the molecular level. For example, the study by Lumba *et al*. [2], which identifies a role for ethylene in regulating vegetative phase transitions, employed the pharmacological levers aminoethoxyvinylglycine (AVG), an inhibitor of ethylene biosynthesis, and silver, an inhibitor of ethylene perception that binds directly to the ethylene receptors. Among the genetic levers employed were the mutations *ein2*, which confers ethylene insensitivity, and *eto1*, which results in mis-regulation of the ethylene biosynthetic pathway so that the plant produces excess ethylene.

## Tailoring the ethylene signal to regulation of particular developmental pathways

If ethylene responses all rely upon the same primary signaling pathway, how is it that ethylene can mediate so many different developmental responses? The answer is likely to rely to a large part on the extent to which transcriptional output from the ethylene signaling pathway can be tailored to meet different needs. Multiple mechanisms have been identified by which such regulation can be accomplished. As explained below, these mechanisms include physical interactions among transcriptional regulators, cooperative control of developmental pathways, and the modulation of expression and protein turnover of elements within the transcriptional cascade.

Two recent studies highlight how physical interactions between the EIN3-like transcription factors and other transcriptional regulators can modulate gene expression. The hormones ethylene and jasmonic acid often act synergistically in the control of plant development and pathogen responses, and a mechanistic basis for this synergy was uncovered when it was discovered that JAZ repressor proteins physically interact with and inhibit the activity of the EIN3-like transcription factors [6]. Signaling by jasmonic acid stimulates the degradation of the JAZ repressor proteins, thereby activating transcription by the EIN3-like transcription factors. The second study identified an interaction between the EIN3-like transcription factors and the transcription factor FIT, a regulator of iron uptake in roots [7]. Protein levels of both the EIN3-like transcription factors and FIT are regulated by proteasome-mediated degradation. In the presence of ethylene, the EIN3-like transcription factors are stabilized, bind to FIT, and apparently protect FIT from degradation, thereby enhancing its ability to stimulate the expression of genes involved in iron acquisition.

The EIN3-like transcription factors can also function cooperatively with other transcription factors to promote developmental changes. This ability is highlighted in a study examining how the EIN3-like transcription factors and the basic helix-loop-helix (bHLH) transcription factor PIF1 play roles in promoting the greening of seedlings exposed to light [8]. Both regulate chlorophyll biosynthesis but apparently do so through the targeting of different promoter elements: the EIN3-like transcription factors target the promoters of *PORA *and *PORB *while PIF1 targets the promoter of *PORC*. These three *POR *genes encode different isoforms for a rate-limiting enzyme in chlorophyll biosynthesis. Thus, PIF1 and the EIN3-like transcription factors function in parallel to facilitate the same developmental pathway.

Not all regulation is at the level of the EIN3-like transcription factors, and downstream ERF and EDF transcription factors greatly increase the potential points for interaction and cross-talk of ethylene signaling with other pathways. For instance, overexpression of the MADS-Box gene *FYF *inhibits floral organ abscission and senescence in *Arabidopsis*, an effect correlating with a decreased responsiveness of the transgenic plants to ethylene [9]. However, no changes were uncovered in the expression of genes encoding elements of the primary signal transduction pathway for ethylene. Instead, the apparent cause of the phenotype is a decrease in the expression of *EDF*s. Furthermore, in monocots, the *FRIZZY PANICLE *(*FZP*) gene of rice and the orthologous *BRANCHED SILKLESS1 *(*BD1*) gene of maize both encode ERF transcription factors, and their loss results in plants that fail to make the transition from inflorescence meristems to floral meristems [10].

The study by Lumba *et al*. [2] suggests that several of these regulatory mechanisms play roles in how ethylene regulates a developmental pathway for advancing juvenile to adult leaves. The leaves of *Arabidopsis *exhibit a gradient of morphological characteristics - involving such attributes as size, shape, and the presence of trichomes - that is dependent on when the leaves are produced during plant development. These characteristic features have facilitated the identification of heterochronic mutations that affect the timing of leaf development, such as mutations in *FUSCA3 *(*FUS3*) resulting in juvenile leaves that have more adult-like traits. FUS3 is a transcription factor and prior work has demonstrated that its effect on leaf phase transitions involves the plant hormones abscisic acid and gibberellin, with abscisic acid retarding and gibberellin advancing the transition from juvenile to adult leaf morphology [3]. Now a third hormone, ethylene, is thrown into the mix [2]. Interestingly, ethylene is here found to stimulate a much earlier stage of development than it is normally associated with, accelerating the transition from juvenile to adult in newly formed leaves, but it is nevertheless still acting as an 'aging' hormone. A principal role of FUS3 appears to be to inhibit ethylene-regulated signal output, as its loss results in increased levels of the EIN3 protein as well as increased expression of *ERF*s and *EDF*s. Part of this effect of ethylene-regulated gene expression is owed to the action of FUS3 on the primary ethylene response pathway. However, at least part of the effect of FUS3 appears to be ethylene-independent as the presence of putative FUS3 binding elements in promoters of ethylene responsive genes raises the possibility that FUS3 also plays a more direct and antagonistic role in controlling transcriptional output from the ethylene signaling pathway. It is likely that similarly complex mechanisms, operating at multiple points in the ethylene signaling pathway, will be uncovered as we begin to explore how ethylene interacts with other plant developmental pathways in more detail.
